# Optimal Information Representation and Criticality in an Adaptive Sensory Recurrent Neuronal Network

**DOI:** 10.1371/journal.pcbi.1004698

**Published:** 2016-02-16

**Authors:** Oren Shriki, Dovi Yellin

**Affiliations:** 1 Department of Cognitive and Brain Sciences, Ben-Gurion University of the Negev, Beer-Sheva, Israel; 2 Department of Computer Science, Ben-Gurion University of the Negev, Beer-Sheva, Israel; 3 Zlotowski Center for Neuroscience, Ben Gurion University, Beer-Sheva, Israel; 4 Department of Neurobiology, Weizmann Institute of Science, Rehovot, Israel; Indiana University, UNITED STATES

## Abstract

Recurrent connections play an important role in cortical function, yet their exact contribution to the network computation remains unknown. The principles guiding the long-term evolution of these connections are poorly understood as well. Therefore, gaining insight into their computational role and into the mechanism shaping their pattern would be of great importance. To that end, we studied the learning dynamics and emergent recurrent connectivity in a sensory network model based on a first-principle information theoretic approach. As a test case, we applied this framework to a model of a hypercolumn in the visual cortex and found that the evolved connections between orientation columns have a "Mexican hat" profile, consistent with empirical data and previous modeling work. Furthermore, we found that optimal information representation is achieved when the network operates near a critical point in its dynamics. Neuronal networks working near such a phase transition are most sensitive to their inputs and are thus optimal in terms of information representation. Nevertheless, a mild change in the pattern of interactions may cause such networks to undergo a transition into a different regime of behavior in which the network activity is dominated by its internal recurrent dynamics and does not reflect the objective input. We discuss several mechanisms by which the pattern of interactions can be driven into this supercritical regime and relate them to various neurological and neuropsychiatric phenomena.

## Author Summary

The recurrent interactions among cortical neurons shape the representation of incoming information but the principles governing their evolution are yet unclear. We investigate the computational role of recurrent connections in the context of sensory processing. Specifically, we study a neuronal network model in which the recurrent connections evolve to optimize the information representation of the network. Interestingly, these networks tend to operate near a "critical" point in their dynamics, namely close to a phase of "hallucinations", in which non-trivial spontaneous patterns of activity evolve even without structured input. We provide insights into this behavior by applying the framework to a network of orientation selective neurons, modeling a processing unit in the primary visual cortex. Various scenarios, such as attenuation of the external inputs or increased plasticity, can lead such networks to cross the border into the supercritical phase, which may manifest as neurological and neuropsychiatric phenomena.

## Introduction

The anatomical abundance of lateral interactions [1, 2] between neurons of the local cerebral circuit (referred in this text as recurrent connections) suggest they play a fundamental role in cortical function. Indirect physiological evidence of their involvement in memory [3, 4], sensory processing [5] and in other brain functions [6, 7] reinforces this notion. Various models have been put forward in an attempt to explain the role of these lateral connections, however, an agreed framework is still missing and the topic is still far from being concluded. In the narrower scope of early visual cortex, some studies have related the role of recurrent connectivity to orientation tuning and contrast invariance [8–10]. Others have suggested a role in generating the accurate firing rates common to spontaneous activity [11].

An additional aspect of recurrently connected networks (relative to networks connected by feedforward links only) involves their dynamic properties. Networks with recurrent connections have been shown to form associative-memory related attractor states[12, 13], exhibit self-organization leading to “neuronal avalanches” [14, 15], and in general, have the potential to exhibit critical dynamics [16–18]. The idea that brain areas may operate near criticality was proposed on theoretical grounds by several authors in the past [18–22]. There is also a growing bulk of recent experimental evidence supporting it [14, 15, 23–26] (for reviews on near criticality in the brain see [16, 27]). Beggs and Plenz [14, 15] demonstrated that neural activity in acute slices and in slice cultures is organized in *neural avalanches*, whose size obeys a power law distribution. They interpreted their results in terms of *critical branching processes* [28]. Further work [23] showed that neuronal avalanches also appear in the spontaneous cortical activity of awake monkeys and in large scale human brain activity (e.g. [29, 30]). It was also demonstrated in slice cultures that the dynamical range of the network is maximized near the critical point [24]. Although these dynamic properties have by now been well established, only few papers in the neuroscience literature have so far attempted to link them to concrete brain functions, such as the function of the visual system.

A central question regarding recurrent interactions, which has not yet been properly addressed, is how they evolve to facilitate the network’s computational capacity and what principles govern this evolution. Their optimal pattern within the network also remains unknown. In this work, we address these issues using a first-principle information theoretic approach, namely using the principle of maximum information preservation (also known as ‘infomax’ [31]). This principle has been successfully implemented in a variety of computational neuroscience studies. Bell & Sejnowski [32] extended it to nonlinear output neurons implementing ICA (Independent Component Analysis) to achieve blind source separation. Later, they showed that the independent components of natural scenes are Gabor-like edge filters [33].

Tanaka et al [34] have demonstrated that the characteristics of orientation selectivity in V1 can be acquired by self-organization of recurrent neural networks according to Infomax learning. This work was recently extended by Hayakawa et al [35] to reveal a biologically plausible infomax learning algorithm.

The present work can be seen as a further extension of these earlier efforts, studying how the gradual development of a network’s recurrent interactions may optimize the representation of input stimuli. Unsupervised learning is applied in training networks to maximize mutual information between the input layer and an overcomplete recurrently connected output layer. The evolving pattern of recurrent interactions is investigated in a model of a hypercolumn in primary visual cortex, considered the base functional unit of V1, receiving input from both eyes, in a full representation of all possible orientations. Various constellations of input stimuli and network connectivity are examined, in aim of studying their relationship with different network measures. Methods to evaluate the optimal pattern of recurrent interactions in a neural network model and its dependence on the statistics of the external inputs were extended from Shriki et al. [36]. We first provide an analytical and numerically simulated account of a toy hypercolumn network model. Subsequently, a more ecological network is studied, in which natural scenes are used as input for training the network. These models allow us to compare the emerging network’s properties with those arising from earlier empirical and theoretical work.

## Methods

The general scheme and many methods applied in this study can be viewed as a direct evolution of the earlier work reported in [36]. Below, we highlight the main extensions of the current models relative to the one presented in this former work in regards to the network structure, learning algorithm and other significant model ingredients.

### Network architecture and dynamics

**Fig 1:**
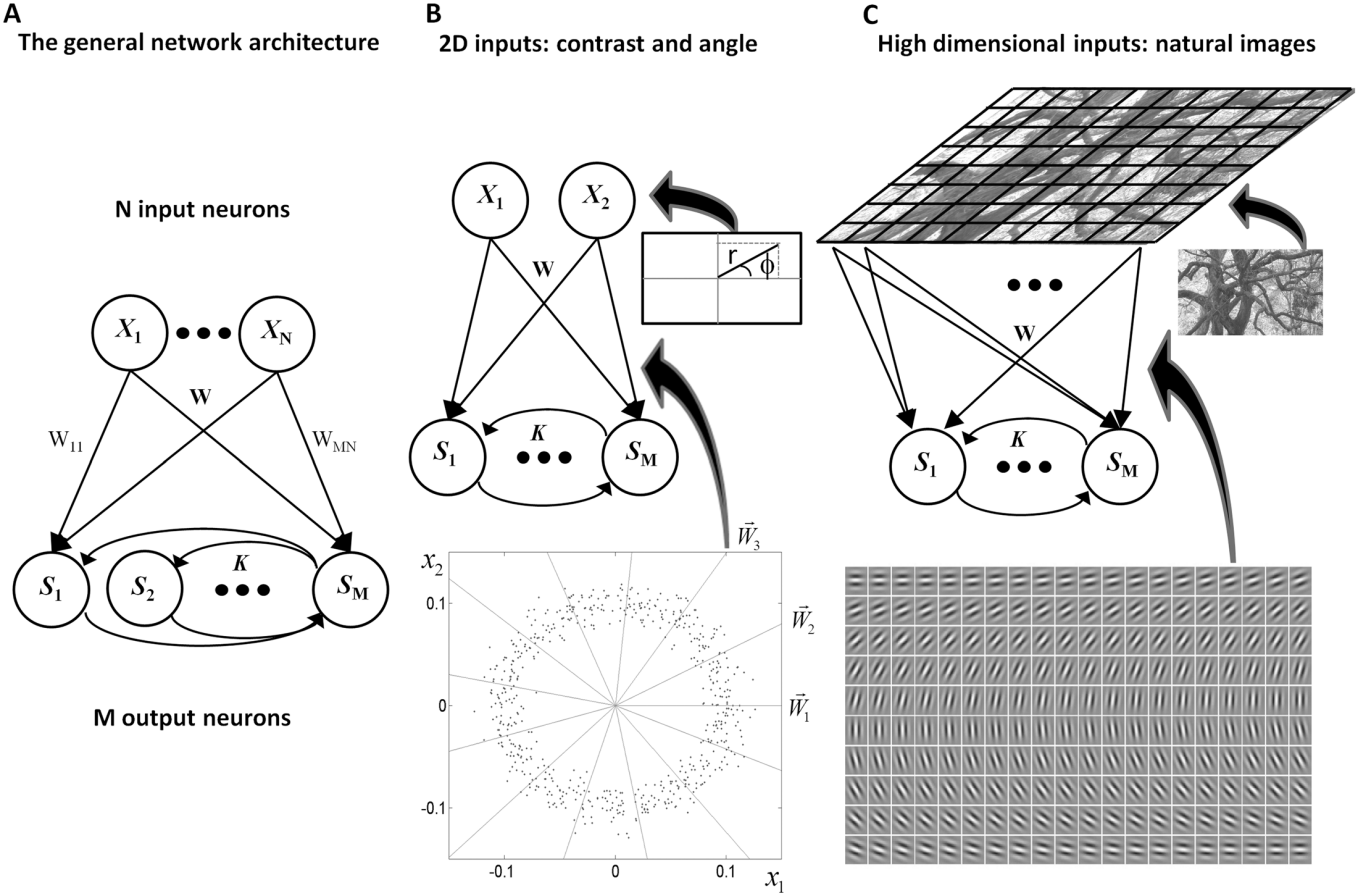
Network diagrams and input statistics. (A) The general network architecture characterized by an overcomplete representation with *N* input neurons, *x*_*i*_ (*i* = 1,…,*N*) and *M ≥ N* output neurons, *s*_*i*_ (*i* = 1,…,*M*). The input is linearly transformed by the feedforward connection matrix,***W***, and then nonlinearly processed by the recurrent dynamics at the output layer. The interactions among the output neurons are denoted by the matrix ***K***. (B) A toy network model of a visual hypercolumn containing 2 input neurons and *M* output neurons. The feedforward connections are preset to unit vectors spanning all angles at equal intervals. The inputs are points on the plane with uniformly distributed angles and normally distributed distances from the origin. The distance from the origin represents the input contrast. (C) An ecological network model in which the inputs are natural images. The feedforward connections are Gabor filters with orientations equally spaced between 0° and 180°.

The basic network model consists of two layers of neurons, *N* neurons at the input layer and *M* neurons at the output layer (Fig 1A), where *M ≥ N*. Thus, the network deterministically maps a low dimensional input space into a manifold in a higher-dimensional output space. Such a representation, which contains more output components than input components, is termed *overcomplete*. The feedforward interactions are described by the *M* × *N* matrix *W* and the recurrent interactions by the *M* × *M* matrix *K*.

During the presentation of each input sample, the input components *x*_*i*_ are fixed. The dynamics of the output neurons are given by

$$\tau \frac{ds_{i}}{dt}=-s_{i}+g\left(\Sigma_{j=1}^{N}W_{ij}x_{j}+\Sigma_{k=1}^{M}K_{ik}S_{k}\right),\;i=1,\ldots ,M$$ (1)

Where *g* is some nonlinear squashing function and *τ* is a characteristic time scale (here we set *τ =* 1 and *g* was taken to be the logistic function, *g*(*x*) = 1/(1+*e*^−*x*^)). We assume that the activities of the output neurons reach equilibrium after some time and define the output as the steady-state pattern of activity. For the cases we studied, numerical simulations of the network dynamics indeed stabilized and proved this assumption to be consistent. The steady-state responses are given by

$$s_{i}=g\left(\Sigma_{j=1}^{N}W_{ij}x_{j}+\Sigma_{k=1}^{M}K_{ik}S_{k}\right),\;i=1,\ldots ,M$$ (2)

### Objective function and learning algorithm

To evaluate the neuronal representation of the external inputs we used the mutual information between the input and output of the network [37]. More specifically, the mutual information between the input vector, ***x***, and the output vector, ***s***, can be expressed as the difference between the entropy of the output and the conditional entropy of the output given the input. The conditional entropy can also be viewed as the entropy of the output noise. Here, the network response is a deterministic function of the input, and thus the mutual information depends only on the entropy of the outputs. As shown in [36], maximizing the output entropy (and therefore the mutual information) is equivalent to minimizing the following objective function:

$$\begin{array}{l} \varepsilon =-\frac{1}{2}{\langle \text{ln det}\left(\chi^{\text{T}}\chi \right)\rangle }_{x}=-\frac{1}{2}\text{Tr}{\langle \text{ln}\left(\chi^{\text{T}}\chi \right)\rangle }_{x} \end{array}$$ (3)

where $x_{ij}=\frac{\partial s_{i}}{\partial x_{j}}$ is the Jacobian matrix of the transformation and reflects the sensitivity of the output units to changes in the input units. We also refer to this matrix as the susceptibility matrix as it is analogous to the susceptibility of physical systems to external fields.

The adaptive parameters of the algorithm are the sets of feedforward and recurrent interactions, *W*_*ij*_ and *K*_*ij*_. The learning rules for these parameters are derived from this objective function using the gradient decent method, as shown in [36]. Here we focus only on the recurrent interactions. The gradient descent learning rule for the recurrent interactions is:

$$\Delta K=-\eta \frac{\partial \varepsilon }{\partial K}=\eta \langle {\left(\chi \Gamma \right)}^{T}+\phi^{T}as^{T}\rangle$$ (4)

Where *η* is the learning rate, the matrix *ϕ* is given by *ϕ* = (*G*^−1^−*K*)^−1^ and satisfies χ = *ϕW*, the matrix *G* is defined as ${\text{g'}}_{k}\;\to {\text{ g'}}_{i}$, the matrix Γ is defined as Γ = (*χ*^*T*^*χ*)^−1^*χ*^*T*^*ϕ* and the components of the vector *a* are given by $a_{k}={\left[\chi \Gamma \right]}_{kk}\frac{g_{k}^{\prime \prime }}{{\left(g_{k}^{\prime }\right)}^{3}}$. The triangular brackets denote averaging over the input samples.

### Metrics of network behavior

We defined several measures to characterize the behavior of the network and gain further insight into its dynamics. As described in the Results section, after the learning process converges, the networks tend to operate near a critical point. Thus, it is helpful to define metrics that may behave differently when the networks approach that critical point. One such measure is the time it takes the recurrent network dynamics to reach steady-state—the *convergence time*. Many dynamical systems exhibit a slow-down of the dynamics near critical points, often termed *critical slowing down* [38]. Thus, a substantial increase in the convergence time may indicate that the system is close to a critical point.

To gain insight in the present context, we note that near a steady state, the linearized dynamics (in vector notation) are given by $\;\tau \frac{d\left(\delta s\right)}{dt}=-\left[I-GK\right]\delta s$. The inverse of the matrix [*I*−*GK*] appears also in the expression for the Jacobian matrix, which determines the objective function. Optimizing the objective function leads to very large eigenvalues in the Jacobian matrix (high-susceptibility), and therefore, the eigenvalues that dominate the dynamics become very small, which manifests as slowing down.

To estimate the convergence time, we defined a criterion for stability of the neuronal activities and measured the time it takes the network to satisfy this criterion. This stability criterion means that for each neuron in the network, the difference in its activity between the current time step and the previous time step is smaller than a predefined small number.

When the network becomes supercritical, it converges onto attractor states, which reflect the underlying connectivity. In the context of orientation tuning, which we study here, a natural measure to quantify this behavior is the population vector [39]. Each neuron is associated with a complex number. The magnitude of the number is the activity of this neuron and the phase is set according to the preferred angle or orientation of the neuron (in the case of preferred orientation, the orientation is multiplied by 2, to span the range from 0° to 360°). Given a pattern of activity in the network, these complex numbers are summed to yield a resultant complex number, termed the population vector. When the network response is uniform, the magnitude of the population vector is 0. When the network response peaks at some orientation, the magnitude of the population vector is finite.

### Training using natural images

Similar to previous papers concerning training of networks over natural scenes [33], we used photos involving forest scenes or single trees and leaves. The photos were converted to grayscale byte value of 0 to 255 and then”cut” into patches of 25-by-25 pixels. Each patch was represented as a vector with 625 components. Using PCA (Principal Component Analysis), the dimensionality of the images was reduced from 625 to 100. The inputs were also whitened by dividing each eigenvector by the square root of the corresponding eigenvalue. These whitened 100-dimensional inputs were used to train a network with 380 output neurons. The results were robust to different manipulations of the inputs. For example, we obtained qualitatively similar results even without dimensionality reduction or whitening, using smaller image patches.

The feed-forward filters were set to be Gabor filters with the same center in the visual field and the same spatial frequency. The size of each Gabor filter was 25-by-25 pixels. The full feed-forward matrix was a product of two matrices: A 380-by-625 matrix containing a Gabor filter in each row, which was multiplied from the right by a 625-by-100 matrix representing the reconstruction after the dimensionality reduction.

### Running simulations in practice

Close to the critical point, accurate simulation of the network dynamics requires a long time due to the phenomenon of *critical slowing down*. To explore the characteristics and dynamics of the network as it approached the critical point, we allowed simulations to run for very long periods. Thus, simulations could take up to weeks to complete based on network size and the value of the learning rate.

When the evolving networks approached a critical point, the objective function tended to be very sensitive to changes in the pattern of interactions. In some cases, the objective function could even increase rather than decrease, implying that the learning rate was not small enough. To overcome this problem, we calculated the expected value of the objective function before actually updating the interactions. When an upcoming increase was identified, the learning rate was reduced by a factor of one-half and the process was repeated again.

## Results

To establish the credibility of our model, we first identified conditions under which a comparison between analytical and numerical results could be facilitated. This was achieved via a toy model of a visual hypercolumn, which is amenable to analytical solution in the limit of very low contrast. An important insight from this toy model is that in the low contrast limit, optimal information representation is obtained at a critical point of the network dynamics. These results are then verified using numerical simulations of this simple model. Using similar simulation approach, we next show that critical behavior also arises in a more complex setting, when natural images are used as inputs in the training phase.

### Infomax and criticality: Insights from analytical and numerical solution of a toy model

The architecture of the network model is presented in Fig 1B. Each input sample is a point on the plane, with an angle, *θ*_0_, representing the orientation of a visual stimulus and amplitude (its distance from the origin), *r*, representing the timulus contrast (Fig 1B). Each point can be represented as (*x*_1_,*x*_2_) = r(cos*θ*_0_,cos*θ*_0_). For clarity, we consider periodicity of 360° rather than 180°, which is the relevant symmetry when considering orientations. The angles *θ*_0_ are distributed uniformly between 0 and 2*π*. The amplitudes *r* are distributed according to a Gaussian distribution with a positive mean 〈*r*〉, representing the mean contrast. By varying the mean value of *r* we study the effect of stimulus statistics on the optimal network connections.

The network represents this two-dimensional input by *M* sigmoidal neurons (*M*≫1) interconnected with recurrent interactions (*K*_*ij*_, *i*,*j* = 1,…,*M*). The feedforward connections (rows of) are chosen to be unit vectors, uniformly distributed over all possible directions, i.e. (*W*_*i*1_,*W*_*i*2_) = r(cos*ϕ*_*i*_,cos*ϕ*_*i*_) where *ϕ*_*i*_ = 2π*i*/*M*, *i* = 1,…,*M*. Thus, the input to the *i*’th neuron has a cosine tuning function peaked at *ϕ*_*i*_ and the network has a ring architecture (Fig 1B). The feedforward connections are fixed throughout the learning. Our goal is to evaluate the matrix of recurrent connections *K* that maximizes the mutual information between the steady state responses of the output neurons and the stimulus. For a given input and connection matrix, the steady-state responses are given by

$$s_{i}=g\left(\Sigma_{j}K_{ij}s_{j}+x_{1}\text{cos}\theta_{i}+x_{2}\text{sin }\theta_{i}\right)$$ (5)

where *g* is the logistic function (see Methods).

The sensitivity matrix, *χ*, is an *M*×2 matrix given by:

$$\chi_{i1}=\frac{\partial s_{i}}{\partial x_{1}}=g_{i}^{\prime }\cdot \left[\Sigma_{l}K_{il}\chi_{l1}+\text{cos}\theta_{i}\right]$$ (6)

$$\chi_{i1}=\frac{\partial s_{i}}{\partial x_{2}}=g_{i}^{\prime }\cdot \left[\Sigma_{l}K_{il}\chi_{l2}+\text{sin}\theta_{i}\right]$$ (7)

Where $g_{i}^{'}=g'\left(\Sigma_{j}K_{ij}s_{j}+x_{1}\text{cos}\theta_{i}+x_{2}\text{sin }\theta_{i}\right)$ is the derivative function of the neuronal transfer function and we have used the expression for *s*_*i*_ given in Eq (5).

To investigate analytically the optimal pattern of recurrent interactions when the typical input contrast is low, namely when 〈*r*〉→0, we assume that the interaction *K*_*ij*_ between the *i*’th and *j*’th neurons is an even function of the distance between the neurons on the ring,

$$K_{ij}=K\left(\theta_{i}-\theta_{j}\right)$$ (8)

When 〈*r*〉 approaches zero, the total external input to each neuron approaches zero. We denote the value of *g*’ at zero input by *γ*_0_ = *g*’(0). In the case of the logistic function, *γ*_0_ = 1/4. Since the number of output neurons, *M*, is large, we can take the continuum limit and transform the summations over angles to integrals. For instance, the equation for *χ*_i1_ can be written as

$$\chi_{1}\left(\theta \right)=\gamma_{0}\left[\frac{M}{2\pi }\int_{-\pi }^{\pi }d\theta^{\prime }K\left(\theta -\theta^{\prime }\right)\chi_{1}\left(\theta \prime \right)+\text{cos}\theta \right]$$ (9)

and similarly for *χ*_i2_. We define the Fourier series of *K* and *χ*_1_

$$K\left(\theta \right)=\frac{1}{M}\Sigma_{n=0}^{\infty }k_{n}\text{cos}\left(n\theta \right)$$ (10)

$$\chi_{1}\left(\theta \right)=\Sigma_{n=0}^{\infty }\left[a_{n}\text{cos}\left(n\theta \right)+b_{n}\text{sin}\left(n\theta \right)\right]$$ (11)

Fourier transforming Eq (9) yields $a_{n}=\gamma_{0}\delta_{n1}/\left(1-\frac{1}{2}\gamma_{0}k_{1}\right)$ and *b*_*n*_ = 0, where *k*_1_ is the first cosine harmonic of the interaction profile, Eq (10). Thus,

$$\chi_{i1}=\frac{\gamma_{0}\text{cos}\theta_{i}}{1-\frac{1}{2}\gamma_{0}k_{1}}$$ (12)

and similarly

$$\chi_{i2}=\frac{\gamma_{0}\text{sin}\theta_{i}}{1-\frac{1}{2}\gamma_{0}k_{1}}$$ (13)

The 2 X 2 matrix *χ*^*T*^*χ* is a diagonal matrix with elements

$${\left(\chi^{\text{T}}\chi \right)}_{11}={\left(\chi^{\text{T}}\chi \right)}_{22}=\frac{M\gamma_{0}}{2{\left(1-\frac{1}{2}\gamma_{0}k_{1}\right)}^{2}}$$ (14)

Substituting these expressions in Eq (3), yields

$$\varepsilon =\text{log}\left(\frac{2}{M\gamma_{0}^{2}}\right)+2\text{log}\left(1-\frac{1}{2}\gamma_{0}k_{1}\right)$$ (15)

Eq (15) implies that as *k*_1_ approaches the critical value $k_{1}^{c}=$ 2/*γ*_0_ the objective function diverges to −∞. This means that the optimal pattern of recurrent interactions has the form

$$K\left(\theta_{i}-\theta_{j}\right)=\frac{2}{M\gamma_{0}}\text{cos}\left(\theta_{i}-\theta_{j}\right)$$ (16)

The divergence of the objective function, that is of the sensitivity (or susceptibility) at $k_{1}^{c}$ reflects the fact that at this point the network undergoes a phase-transition into a state of *spontaneous symmetry breaking* [9]. Formally, this can be illustrated by adding a uniform random component to the input that each neuron receives and examining the network response. As shown in [9], the network response is very different below and above the transition point. For $k_{1}^{c}$<2/*γ*_0_, the network settles into a homogeneous state with *s*_*i*_ = *g*(0). However, for $k_{1}^{c}$>2/*γ*_0_, the network dynamics evolve into an inhomogeneous solution with a typical ''hill'' shape [9], which is determined by the recurrent connections and can be interpreted as a "hallucination" of an oriented stimulus. Neurons, which are slightly more active due to the random noise, enhance the activity of neurons with similar preferred orientations, which in turn enhance the activity of the initial neurons through feedback. The winning neurons inhibit neurons with more distant preferred orientations, thus creating a "hill"-shaped profile. The location of the peak of this hill is arbitrary and depends on the specific realization of the noise in the input pattern and on the initial conditions of the neuronal activities. This dramatic change in the network behavior implies that near $k_{1}^{c}$ the network is extremely sensitive to small changes in the input. This enhanced sensitivity increases the mutual information between the network response and the stimulus.

In the limit of 〈*r*〉→0 the objective function depends solely on the first harmonics of the interaction profile, leaving open the question of whether the higher order corrections in *r* predict large values of the higher harmonics of the interaction profile. Furthermore, in the analytic derivation we have assumed translational invariance of *K*, which raises the question of whether there are better solutions which break this symmetry of *K*. To address these questions, we simulated the gradient based learning algorithm for the evolution of the interaction matrix (Methods; [36]), with no restrictions on the form of the matrix. The network consisted of 2 input neurons and 141 output neurons. The nonlinear squashing function was the logistic function. The feedforward connections to each output neuron were unit vectors uniformly distributed between 0° and 360°, and were fixed throughout the learning. The initial recurrent interaction matrix was set to zero. The angle of each input was drawn from a uniform distribution, while the magnitude was drawn from a Gaussian distribution around a characteristic radius *r* with a standard deviation of 0.1 times the mean.

**Fig 2:**
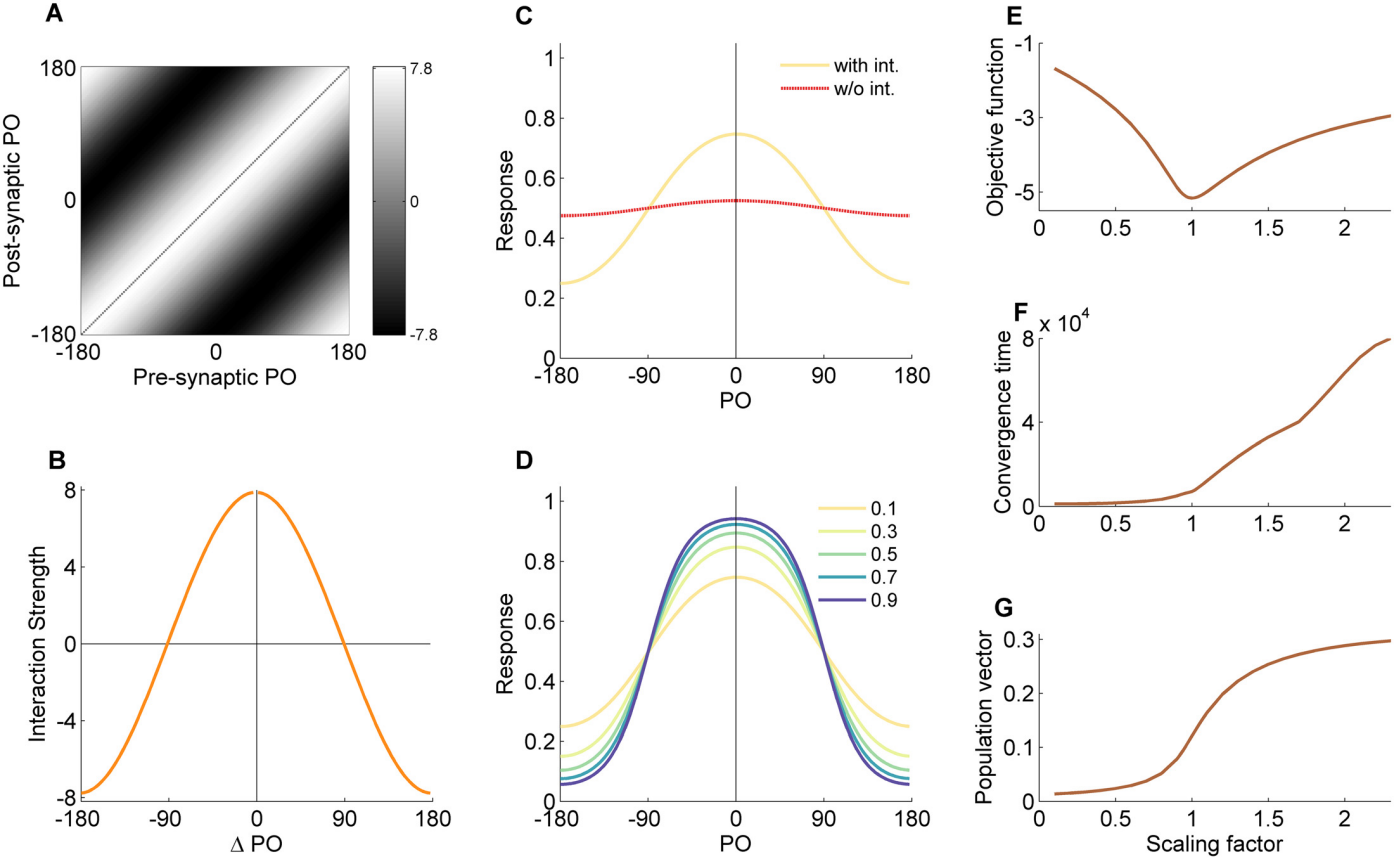
Behavior of the simplified hypercolumn model in the limit of low input contrast. Results of the toy model following gradient-descent learning that minimizes the objective function at a mean contrast of 〈*r*〉 = 0.1. (A) The optimal interaction matrix representing the strength of recurrent connections with gray level value (interaction from neuron *j* onto neuron *i* as grey level of the pixel in the *i’*th row and *j’*th column). (B) The interaction profile for the neuron tuned to 180° (the middle column of the interaction matrix). (C) Network response in presence and absence of recurrent interactions, for an input with contrast of *r* = 0.1. The dashed line is the response of the network without the recurrent interactions and the solid line is the response with them. (D) The network’s response amplification to inputs at different levels of contrast. (E-G) The effect of scaling the recurrent interactions on several metrics of network behavior. (E) Objective function. (F) Convergence time of the recurrent network. (G) Magnitude of the population vector of the network response. PO—preferred orientation.

Fig 2 shows the results from a simulation with 〈*r*〉 = 0.1, namely when the inputs are relatively weak. As can be seen, the interaction pattern (Fig 2A) is translation invariant; i.e., each neuron has the same pattern of pre and postsynaptic interactions. It is important to note that we do not impose any symmetry on the connections. The resulting translation invariance is a natural result of the statistical symmetry of the inputs to the network. Fig 2B shows one row of the interaction matrix (representing the presynaptic connections into a single output neuron). For clarity, the values are multiplied by the number of neurons, *M*. This result is highly congruent with the analytical derivation presented above, Eq (16), that predicts a pure cosine profile with an amplitude of 8 for the logistic function. Fig 2C shows the response of the network as a function of the preferred orientation (PO) of the neurons (solid line) to a vertical input at the typical contrast (*r* = 0.1). The amplification in comparison to the network response without recurrent interactions (dashed line) is clearly seen. Responses to different contrasts are shown in Fig 2D.

#### Effect of scaling the interactions

While running the numerical simulations, we noticed that the basic shape of the interaction profile appeared already at early stages of the training. During the rest of the learning process, the main factor that changed was the scale of the profile until it reached an optimal value. In this sense, although there were $M^{2}-M$ free parameters, most of the learning took place along a single dimension in the parameter-space. Motivated by this observation, we changed the scale of the optimal recurrent interaction matrix and explored the network behavior as a function of the scaling factor (Fig 2E–2G). Fig 2E depicts the objective function. The fact that it attains its minimum when the scaling parameter is 1, simply means that the optimal scaling is obtained for the optimal interaction matrix that was obtained from the learning process. This is an indication that the learning process indeed converged. The convergence time of the recurrent network dynamics (Fig 2F; Methods) increases substantially near a scaling parameter of 1, indicative of critical slowing down.

Fig 2G shows that the magnitude of the population vector transitions into relatively large values near a scaling parameter of 1. This reflects the fact that above 1 the network dynamics are dominated by hill-shaped attractor states [9]. Overall, the behavior of the convergence time and the population vector shows that indeed close to the optimal scaling factor from the learning process, the network experiences a phase transition.

**Fig 3:**
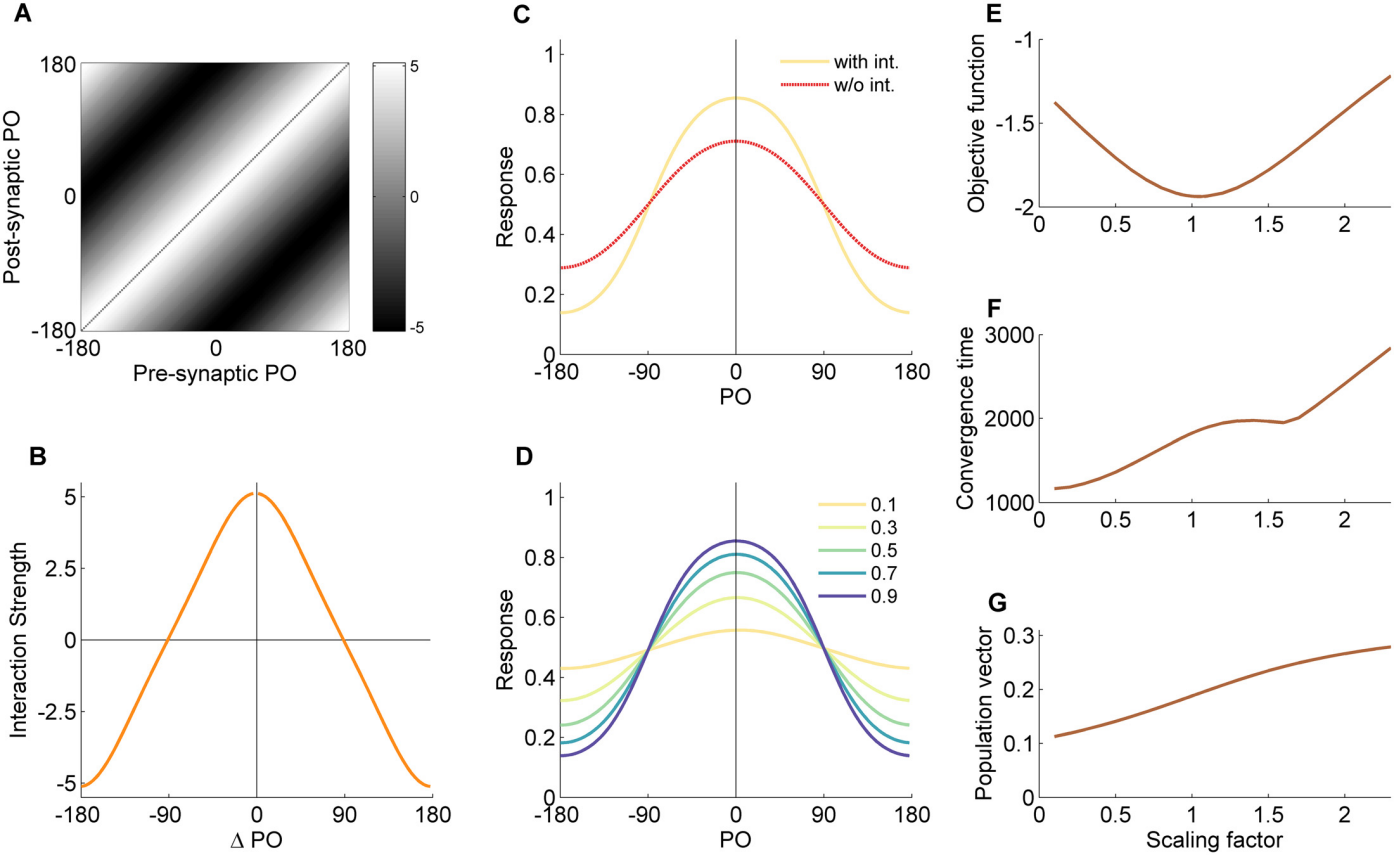
Behavior of the simplified hypercolumn model for intermediate input contrast. Results of the toy model following gradient-descent learning that minimizes the objective function at a mean contrast of 〈*r* 〉 = 0.9. The figure is organized similarly to Fig 2. (A) Interaction matrix. (B) Interaction profile. (C) Network response with and without recurrent interactions. (D) Response amplification at different contrast levels. (E-G) Objective function (E), convergence time (F) and magnitude of the population vector (G) as a function of the scale of the recurrent interactions. Relative to the case of learning in the domain of low input contrasts, here, recurrent interactions are less crucial for performance. PO—preferred orientation.

When the mean input contrast during learning is not too low and not too high the recurrent interactions are less crucial for network performance. Fig 3 depicts the results from a numerical simulation with 〈*r* 〉 = 0.9, namely with an intermediate level of contrast. The interaction matrix (Fig 3A) resembles the one for 〈*r* 〉 = 0.1, but the amplitude of the interaction profile is lower (~5) compared with the low contrast case (~8) and the profile contains higher harmonics. The effect of the recurrent interactions on the network response is less pronounced too (Fig 3C). Fig 3E–3G depict the network behavior when the recurrent interactions are scaled. The network dynamics converge on shorter time scales (Fig 3F) and the magnitude of the population vector does not show a sharp transition (Fig 3G).

### Training with natural images

We next investigated a more complex network model of a visual hypercolumn (Fig 1C). In this setting, gray-level image patches from natural scenery (see Methods) were used as inputs to train the network [40]. The network consisted in this case of 100 input neurons and 380 output neurons. To study the pattern of recurrent interactions systematically, we manually set the feed-forward filters to be Gabor filters with the same center in the visual field and the same spatial frequency, spanning all orientations. It is worth noting that this overcomplete network can also be used to learn the feed-forward connections themselves [36], and indeed, as we established thorough numerical simulations, when trained using natural scenes, the feed-forward filters turn out to be Gabor-like filters. This result is related to the fact that the algorithm for the feed-forward connections is a simple generalization of the infomax ICA algorithm [32] from complete to overcomplete representations. Training the infomax ICA algorithm using natural scenes is known to result in Gabor-like filters [33].

**Fig 4:**
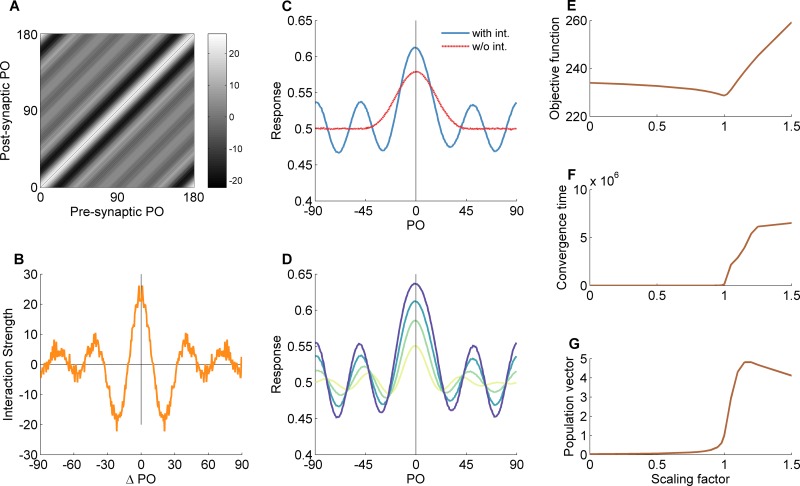
Behavior of the ecological hypercolumn model after training with natural scenes. The figure is organized similarly to Fig 2. (A) Interaction matrix. (B) Interaction profile. (C) Network response with and without recurrent interactions to an oriented stimulus (a Gabor filter with similar properties to the preset feedforward filter). (D) Response amplification at different contrast levels. (E-G) Objective function (E), convergence time (F) and magnitude of the population vector (G) as a function of the scale of the recurrent interactions. Notably, the evolved network dynamics when exposed to natural images resembles more closely the behavior of the toy model after training with low contrast stimuli. PO—preferred orientation.

Fig 4A depicts the full matrix of recurrent connections. As can be seen, the matrix is symmetric and the interaction between two neurons depends only on the distance between their preferred orientations. This finding is in line with the behavior of the simple toy model. Again, it is important to note that the interaction matrix was not constrained to be symmetric. Rather, this is a natural outcome of the learning process, reflecting the symmetry in the pattern of feedforward interactions. Fig 4B plots the interaction strength as a function of the distance between the preferred orientations of the pre- and post-synaptic neurons. The emerging profile has a "Mexican hat" shape, with short-range excitation, longer-range inhibition and an oscillatory decay as the distance in preferred orientation increases.

To characterize the network behavior after training it with natural images we examined its response to simple oriented stimuli. Fig 4C depicts the steady-state profile of activity in response to a vertically oriented Gabor stimulus (solid line). The spatial frequency of the Gabor stimulus and the width of the Gaussian envelope were identical to those of the Gabor filters in the feedforward connections and the contrast was set to the mean contrast of the training stimuli. For comparison, the dashed line shows the response of the network without recurrent interactions. Clearly, the evolved recurrent interactions amplify and sharpen the response compared to the response without recurrent interactions. Fig 4D shows the network response to the same vertical stimulus for various contrast levels. Notably, the width of the profile is approximately independent of the contrast, and the effect of changing the contrast is mainly multiplicative.

Fig 4E–4G show the dependence of various measures for the network behavior (see Methods) on the scaling factor. Fig 4E shows that even small changes to the scale factor can significantly increase the objective function, resulting in poor information representation. Decreasing the scale factor reduces the amplification provided by the recurrent interactions and consequently reduces the sensitivity of the network to external inputs. Conversely, increasing the scale factor to values above 1 causes the recurrent interactions to become too dominant, and pushes the network into a *pattern formation* regime. In this regime, the network is again less sensitive to external inputs, but this time it is due to the attractor dynamics that govern its behavior. Fig 4F shows the convergence time of the network dynamics. At the optimal point, the convergence time starts to increase to very high values, reflecting critical-slowing down at the transition into attractor-dominated dynamics. The magnitude of the population vector also rises sharply near the optimal point (Fig 4G). Overall, the behavior of the convergence time and the population vector shows that indeed close to the optimal scaling factor from the learning process, the network experiences a phase transition. The behavior of these metrics also resembles their behavior in the low contrast case in the toy model (Fig 2F–2G).

## Discussion

We studied the long-term evolution of recurrent interactions in a model of a sensory neural network and their dependence on the input statistics. We found that under very general conditions, optimal information representation is achieved when the network operates near a critical point in its dynamics.

### A first-principle derivation of the pattern of recurrent interactions among orientation columns

The study focused on a simplified model of visual hypercolumn, a local processing unit in the visual cortex. The feedforward interactions from the input layer to the output layer were manually set such that each neuron in the output layer had a certain preferred orientation. The recurrent interactions among these neurons evolved according to learning rules that maximize the mutual information between the external input to the network and the network's steady-state output. When the inputs to the network during learning were natural images, the evolved profile of interactions had a Mexican-hat shape. The idea that neurons with similar preferred orientations should effectively excite each other and that neurons with distant preferred orientations should effectively inhibit each other has been suggested in the past based on empirical findings, e.g. [9, 41, 42], but here it was derived using a first-principle computational approach. This pattern of interactions helps in amplifying the external inputs and in achieving a relatively constant width for the orientation tuning curves, which is consistent with experimental findings on primary visual cortical neurons [43, 44].

A learning algorithm for information maximization in recurrent neural networks was also derived in [34]. The major difference from the current work is that here the information is maximized between the external input and the steady-state output, whereas in [34] the input and output refer to the patterns of activity in the recurrent network at two consecutive time steps. The approach in [34] is aimed at maximizing information retention in the recurrent network, whereas here the focus is on sensory processing and on the representation of the external input. In addition, the neurons in [34] are stochastic binary neurons, whereas the neurons here are deterministic and have a smooth nonlinearity. The network model in [34] was also trained using natural images as external inputs, leading to Gabor-like feed-forward connections, consistent with the findings in [33]. However, the authors do not discuss the structure of the connections *among* the output neurons, so this important aspect cannot be compared with the present work, which focused on recurrent connectivity.

The present model is clearly overly simplified in many aspects as a model of the primary visual cortex. For example, the gradient-based learning rules employed here are likely to be very different from the plasticity mechanisms in the biological system, but the assumption is that they reflect the long-term evolution of the relevant neural system and converge to a similar functional behavior. Despite its simplicity, the model provides a concrete setting for examining the role of recurrent interactions in the context of sensory processing. This leads to general insights that go beyond the context of early visual processing, as we discuss below.

### The importance of near-critical recurrent networks

The dynamics of recurrent networks, like the one studied here, can allow the network to hold persistent activity even when the external drive is weak or absent. The network is then said to display attractor dynamics. In the context of memory systems, attractors are used to model associative memory [45, 46]. Different attractors correspond to different memory states, and the activity patterns that form the basin of attraction of each attractor correspond to various associations of this memory. In the context of early sensory networks, however, the persistent activity at an attractor may correspond to a hallucination. In addition, the flow from different initial patterns to the attractor implies loss of information and insensitivity to changes in the external inputs, and thus may be undesired in the context of sensory processing. An important result of this study is that the evolved networks naturally tend to operate near a critical point, which can be thought of as the border between normal amplification of inputs and hallucinations. In [9], a model of a visual hypercolumn, which is similar to our toy model, was studied analytically. There, the pattern of interactions was assumed to have a cosine profile and it was shown that when the amplitude of the cosine crosses a critical value, the network transitions into an attractor regime. In this regime, the network dynamics evolve into an inhomogeneous solution with a typical ''hill'' shape, which represents a hallucination of an oriented stimulus. Here, the learning algorithm leads the network to operate close to that critical point. Scaling up the resulting pattern of synaptic interactions by a small factor pushes the network into the undesired regime of attractors, namely into hallucinations [47, 48].

This tendency to operate near a critical point can be explained intuitively. The task of the network is to maximize the mutual information between input and output, which amounts to maximizing its sensitivity to changes in the external inputs. The network uses the recurrent interactions to amplify the external inputs, but too strong amplification may generate hallucinations. Thus, the learning process should settle at an optimal point, which reflects a compromise between these two factors. An interesting insight comes from comparing the network to physical systems that may experience phase-transitions in their behavior. A universal property of these systems is that their sensitivity to external influences, or in physical terminology their *susceptibility*, is maximized at the transition point [49]. Our adaptive sensory recurrent networks evolve to operate near a critical point in order to achieve maximal susceptibility and represent information optimally. It is important to note that neural systems respond to a wide range of inputs and that the target of the learning is to find the pattern of interactions that is optimal on average. Under certain conditions, the recurrent interactions may not contribute much to the representation. However, in many cases, especially if the typical inputs have a narrow distribution or tend to be weak, the optimal pattern of recurrent interactions is expected to be near critical. The dominance of low contrasts in natural images is therefore an important factor in driving the pattern of recurrent interactions to be near critical.

There are several important distinctions to be made when comparing previous research [14, 15, 24, 27, 50, 51] on critical brain dynamics with the present study. First, the present work addresses mainly the issues of long-term plasticity and the effect of input statistics, whereas previous modeling works consider mostly networks with random connectivity, which do not adapt to input statistics. Here we demonstrated that near-criticality emerges as a result of directly optimizing a well-defined measure for network performance using a concrete learning algorithm. In addition, an important role is played by the input statistics, and depending on these statistics the network may or may not approach criticality. Moreover, the resulting connectivity matrices are not random and the specific pattern that emerges is crucial for the network performance. We note that in [34] the network can adapt to the statistics of external inputs, but there criticality was demonstrated only when the network evolved without external input. Other studies, such as [52], model plasticity in recurrent neuronal networks, but not in an ecological sensory context.

Second, here the critical point relates to the transition from normal amplification of external inputs to an attractor regime. At the supercritical regime, the network may present inhomogeneous activity patterns but it is not necessarily driven to saturation. In other words, the supercritical regime does not necessarily correspond to an explosive growth of the activity or to epileptic seizures. In the subcritical regime, the representation is faithful to the input and cannot generate hallucinations, but the activity does not necessarily die out. This should be compared with models based on branching processes, in which the supercritical regime generally refers to runaway activity and the subcritical regime refers to premature termination of activity. In the present model, the network may have a branching parameter of 1 in both the subcritical and supercritical regimes. In this sense, the type of criticality presented by this model can be thought of as a subspace within the space of all networks with branching parameter equal to 1. Furthermore, in contrast to [21] and [18], the supercritical regime in the present model does not correspond to chaotic behavior.

The issues raised above call for future experimental and theoretical work aimed at elucidating the effect of input statistics on the approach to criticality and at characterizing the type of criticality that emerges. In particular, future modeling work should consider learning algorithms that optimize information representation in spiking and conductance-based neural networks, which have richer dynamics. An interesting approach to take spike times into account is proposed in [53] but the proposed algorithm is limited to one-layer feed-forward networks. Incorporating short-term plasticity in these models would also be valuable, because networks with short-term plasticity were demonstrated to exhibit robust critical dynamics [22, 45, 46].

### Properties of near-critical recurrent networks

An interesting universal phenomenon that occurs when networks approach the critical point is a change in the effective integration times. As demonstrated here, close to the critical point the time it takes the network to settle after the presentation of an input is considerably longer. This phenomenon is termed *critical slowing down*, [38, 54] and it may serve as a probe to characterize near-critical networks both in models and in experiments (e.g., by examining the power spectrum or by measuring the decay time after a perturbation). It should be pointed out that there is a trade-off between the information representation and the integration time. Near criticality, the output of the recurrent network is more sensitive to change in the inputs, but it takes more processing time. It is reasonable to assume that the brain also takes the processing time into account and not only the quality of the representation. This factor should drive networks in the brain to operate slightly below the critical point, i.e. in the subcritical regime, than would be predicted based on information representation alone.

Clearly, because the neurons in our network are characterized by their firing rates, the network dynamics are not rich enough to display spatiotemporal patterns of activity like neuronal avalanches, synchronized firing or chaotic behavior. Nevertheless, the rate models can often be translated to more realistic conductance-based neuronal networks, which display similar dynamics [55]. In particular, the conductance-based model of a hypercolumn that is investigated in [55] exhibits a critical point similar to the one described here, and the network state is neither synchronized nor chaotic in either side of the critical point.

### Routes to criticality

In real-life biological settings, the pattern of recurrent interactions in a network can be driven into the supercritical 'pattern formation' regime as a result of several possible mechanisms. One possibility is via direct application of certain drugs that increase the effective synaptic efficacy. Bressloff et al. [47, 48] studied the dynamics of a network model of the primary visual cortex. They show that when the network's resting state becomes unstable, the various patterns of activity that spontaneously emerge correspond to known geometric visual hallucinations seen by many observers after taking hallucinogens. They propose that hallucinogens act by scaling the synaptic interactions until instabilities in the network dynamics begin to arise. Our work suggests that due to the network operating not far from the critical point, even a relatively small increase in the scale of the connections may drive it into the supercritical domain.

Another plausible scenario for approaching criticality is through a high degree of plasticity. In numerical simulations of the learning algorithm, an important parameter is the learning rate that controls the step size of the learning dynamics and can be biophysically interpreted as the degree of plasticity [56]. Interestingly, in simulations in which the learning rate was too high, the network did not stabilize at the optimal point near the phase transition but instead crossed it due to the large step size, resulting in poor information representation and hallucinatory behavior. This behavior suggests a potential causal relationship between abnormal neural plasticity and neurological or neuropsychiatric phenomena involving hallucinations, such as schizophrenia.

A third route to criticality is through attenuation of the external inputs. When the external inputs to the network are very weak the recurrent interactions at the output layer compensate by further approaching the critical point. This process increases the effective gain of the network but may lead to instabilities in the network dynamics and to false percepts. For instance, such a mechanism may play a role in the generation of hallucinations as a result of sensory deprivation. An interesting example in this context is *tinnitus*, a persistent and debilitating ringing in the ears [57]. Tinnitus often appears after damage to the hair cells in the ear, mostly by acoustic trauma or by pharmacological agents, such as Salicylate. It was also proposed that plasticity of the central nervous system may play a role in the etiology of Tinnitus [58]. Our model suggests that recurrent networks further along the auditory pathway may try to compensate for the attenuated signals by setting their interactions closer to the critical point. Operating too close to this instability may result in spontaneous activity that is manifested as persistent illusory sounds. The idea that sensory deprivation leads to criticality may also be related to the observation of criticality in slices and cultures [2]. A prediction of the present work would be that highly variable external stimulation will result in networks that are non-critical.

It is also interesting to discuss how a network that became supercritical can return to the normal subcritical regime. In principle, the gradient descent learning algorithm should drive the network to the optimal point even when it is supercritical. However, the learning is based on certain continuity assumptions regarding the mapping of input patterns to output patterns, which may be violated in the supercritical attractor regime. In particular, we assume that there is an invertible continuous mapping between input and output with a well-defined Jacobian matrix. Topologically, the output space may become disconnected with different islands corresponding to different attractor states, making the mapping non-invertible and dis-continuous. Under these conditions, the learning algorithm may not be able to optimize information representation and bring the network back to subcritical dynamics. A similar phenomenon might happen in real brains, preventing the intrinsic learning rules from getting the network back to normal healthy dynamics.

### Conclusion

Our findings suggest that optimal information representation in recurrent networks is often obtained when the network operates near criticality. This is consistent with a growing body of theoretical and experimental literature relating to near criticality in the brain [2, 14, 15, 23, 27, 50, 59, 60]. The uniqueness of the present study is in the rigorous approach to the role of long-term plasticity in approaching criticality and we believe that further research should be dedicated to this issue.
